# Global research trends and future directions in diabetic macular edema research: A bibliometric and visualized analysis

**DOI:** 10.1097/MD.0000000000038596

**Published:** 2024-06-21

**Authors:** Yuanyuan Li, Chuanhong Jie, Jianwei Wang, Weiqiong Zhang, Jingying Wang, Yu Deng, Ziqiang Liu, Xiaoyu Hou, Xuqi Bi

**Affiliations:** a Eye Hospital China Academy of Chinese Medical Sciences, Beijing, China.

**Keywords:** bibliometric, Bibliometrix, CiteSpace, diabetic macular edema, visualized, VOSviewer

## Abstract

**Background:**

Diabetic Macular Edema (DME) significantly impairs vision in diabetics, with varied patient responses to current treatments like anti-vascular endothelial growth factor (VEGF) therapy underscoring the necessity for continued research into more effective strategies. This study aims to evaluate global research trends and identify emerging frontiers in DME to guide future research and clinical management.

**Methods:**

A qualitative and quantitative analysis of publications related to diabetic macular edema retrieved from the Web of Science Core Collection (WoSCC) between its inception and September 4, 2023, was conducted. Microsoft Excel, CiteSpace, VOSviewer, Bibliometrix Package, and Tableau were used for the bibliometric analysis and visualization. This encompasses an examination of the overall distribution of annual output, major countries, regions, institutions, authors, core journals, co-cited references, and keyword analyses.

**Results:**

Overall, 5624 publications were analyzed, indicating an increasing trend in DME research. The United States was identified as the leading country in DME research, with the highest h-index of 135 and 91,841 citations. Francesco Bandello emerged as the most prolific author with 97 publications. Neil M. Bressler has the highest h-index and highest total citation count of 46 and 9692, respectively. The journals “Retina – the Journal of Retinal and Vitreous Diseases” and “Ophthalmology” were highlighted as the most prominent in this field. “Retina” leads with 354 publications, a citation count of 11,872, and an h-index of 59. Meanwhile, “Ophthalmology” stands out with the highest overall citation count of 31,558 and the highest h-index of 90. The primary research focal points in diabetic macular edema included “prevalence and risk factors,” “pathological mechanisms,” “imaging modalities,” “treatment strategies,” and “clinical trials.” Emerging research areas encompassed “deep learning and artificial intelligence,” “novel treatment modalities,” and “biomarkers.”

**Conclusion:**

Our bibliometric analysis delineates the leading role of the United States in DME research. We identified current research hotspots, including epidemiological studies, pathophysiological mechanisms, imaging advancements, and treatment innovations. Emerging trends, such as the integration of artificial intelligence and novel therapeutic approaches, highlight future directions. These insights underscore the importance of collaborative and interdisciplinary approaches in advancing DME research and clinical management.

## 1. Introduction

Diabetic macular edema (DME) represents a critical public health issue due to its role in visual impairment among patients with diabetes.^[1–3]^ Research indicates that over two-thirds of patients with real-world DME experience a visual acuity of less than 0.5, significantly affecting their visual health.^[4,5]^ Although significant strides have been made in understanding DME’s epidemiology, pathophysiology, and treatment options, discrepancies and gaps in current research persist. For instance, while the effectiveness of anti-Vascular Endothelial Growth Factor (anti-VEGF) treatments is well-documented, responses vary among individuals, highlighting the need for alternative strategies and a deeper understanding of disease mechanisms. This study aims to provide a comprehensive bibliometric analysis, encompassing the extensive scope of DME research to elucidate trends, identify significant contributions, and outline evolving frontiers within this field.

DME is a subject of extensive study, with numerous scientific publications covering various aspects, including diagnostic methods, treatment strategies, and advancements in disease pathophysiology. This extensive literature poses a challenge in determining the research focus and frontiers. Therefore, we employed bibliometric analysis to objectively quantify and evaluate academic achievements, collaborations, and the thematic evolution in this field to comprehend the current status, research hotspots, and future trends in DME.^[6–8]^ In this context, collaboration is defined as the joint effort of researchers, institutions, and countries working together to contribute to a collective body of knowledge on DME.

An in-depth analysis of the vast body of literature on DME was conducted, utilizing bibliometric techniques to reveal patterns, trends, and key milestones. Our goal is to provide a comprehensive overview of the global landscape of DME research by exploring citation networks, author collaboration, influential journals, and thematic clusters. In addition, identifying key articles, prolific authors, and emerging topics will contribute to a nuanced understanding of the current state of knowledge and guide future directions in DME research.

## 2. Materials and methods

### 2.1. Data source and search strategy

**Figure 1.**
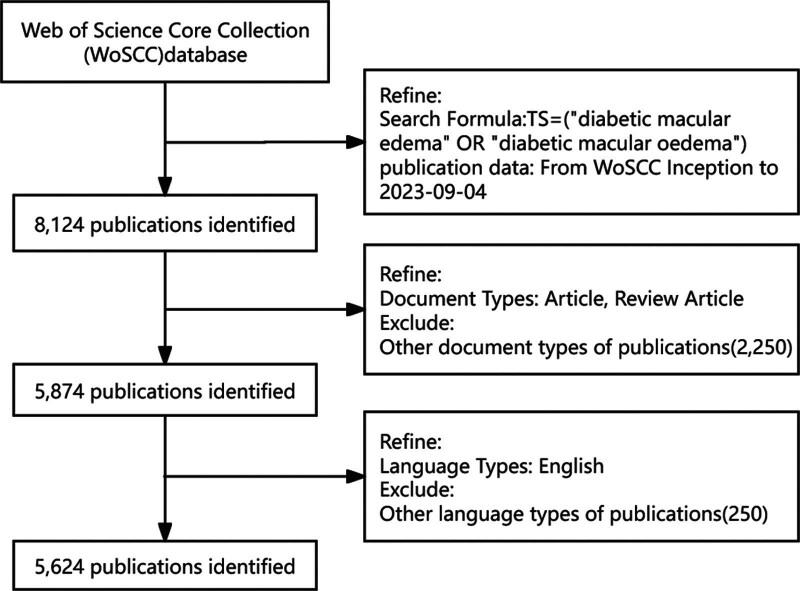
Publication screening flowchart.

The Web of Science Core Collection (WoSCC) was employed to conduct an exhaustive literature search covering DME from its inception to September 4, 2023. The primary search strategy incorporated the following terms: TS = (“diabetic macular edema” OR “diabetic macular edema”). All searches were performed on the same day to maintain consistency and to avoid potential deviations caused by daily database updates. Figure 1 shows the flow chart of study identification and selection.

### 2.2. Eligibility criteria and data collection

The scope of the literature considered in this study was restricted to articles and reviews with a specific focus on English-language publications. Duplicate and unpublished studies were excluded. Identified literature records were systematically exported in batches as plain text files, documented under the category of “Full Record and Cited References.”

A set of key information parameters was systematically gathered from the eligible articles. These parameters encompassed details such as paper count, citation count, title, authorship, geographic location, affiliated research institution, keywords, journal information, publication year, references, impact factor (IF) for the year 2023, h-index, and intensity of citation bursts.

### 2.3. Statistical analysis

The bibliometric data analysis for this study was conducted using the online analysis platform Bibliometric (accessible at http://bibliometric.com/) and various software programs, including Microsoft Excel (version 16.78) and CiteSpace (version 5.7.R5), VOSviewer (version 1.6.19), Bibliometrix (version 4.1.3), and Tableau (version 2023.2.0).

Online analysis tools provided by the WoSCC database and Microsoft Excel (version 16.78) were employed to analyze and visualize bibliometric data related to publications, citations, and the distribution of the h-index across years, countries/regions, institutions, authors, and journals. The h-index, defined as the number of papers published that have been cited h times or more, serves as an indicator of both the quantity and the quality of the academic output of a researcher.^[9]^ Furthermore, the h-index was combined with other indicators to assess the influence of academic research fields, institutions, journals, and papers.

VOSviewer^[10]^ was used to analyze and visualize the bibliometric networks. This software, based on bibliographic datasets, generates various visualization maps, including collaboration maps among authors, institutions, and countries/regions; co-authorship maps indicating the number of coauthors between the most productive sources; keyword co-occurrence maps; and co-citation maps (when 2 papers receive citations from the same article).^[11]^

CiteSpace,^[12]^ developed by Professor Chaomei Chen, is a visual analysis software based on Java. CiteSpace uses hotword analysis to draw knowledge maps reflecting research hotspots and emerging trends at specific stages. In this study, cluster analysis, citation burst analysis, timeline mapping of keywords and references, and a dual-map overlay of citations were performed using CiteSpace.

Tableau 2023.2.0^[13]^ was employed to analyze the global distribution of countries or regions concerning the number of publications and citations.

The Bibliometrix package,^[14]^ a well-established set of tools based on the R language for bibliometric analysis, was used to analyze the evolution of themes, visualize collaboration networks between countries, and construct a three-field plot using Keywords Plus analysis.

Additionally, a bibliometric website was utilized to plot the collaboration analysis and annual publishing data for the countries/regions.

## 3. Results

### 3.1. Overall distribution

The WoSCC database yielded a total of 5624 publications related to DME that met the specified search criteria. Among these, 4790 articles (85.2%) were original research papers, and 834 articles (14.8%) were reviews. The earliest documented report of macular lesions associated with diabetes dates back to 1856 and was authored by Eduard Jaeger.^[15]^ Subsequently, in 1973, Ticho et al^[16]^ published the inaugural article employing the complete term “diabetic macular edema,” focusing on the role of capillary perfusion in DME treatment.

**Figure 2.**
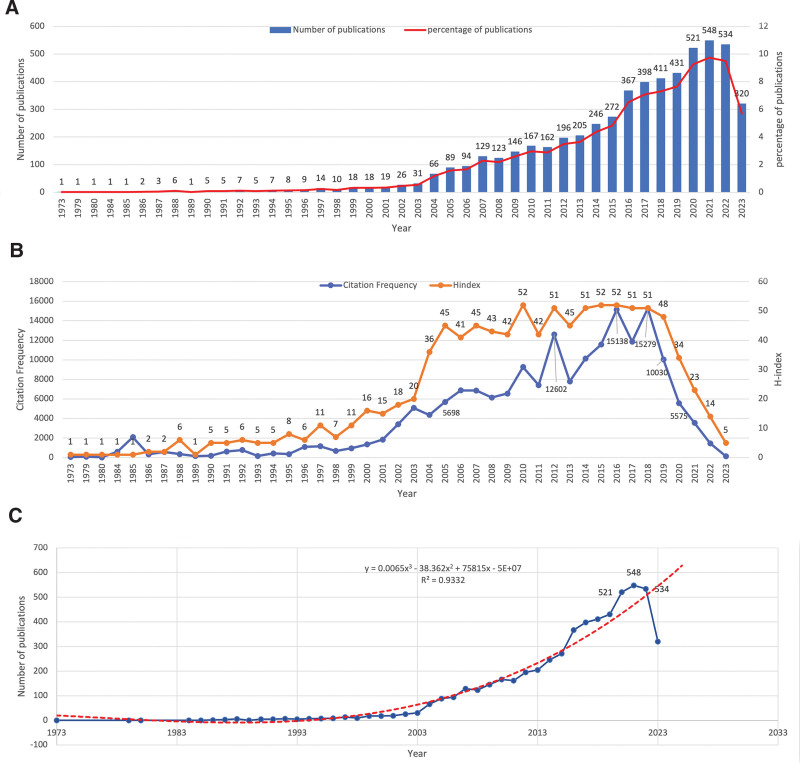
Overall Distribution of publications on diabetic macular edema. (A) Global annual publication distribution. (B) Citation frequency and h-index of the annual publications. (C) Trends of the annual publications.

Prior to 1998, research on DME received limited attention, and published articles were relatively sparse. Post-1998, there has been a steady increase in relevant publications, accompanied by an upward trajectory in citation frequency and the h-index (Fig. 2A and B). This upward trend reflects a growing interest in and focus on DME research, likely influenced by the gradual integration of Optical Coherence Tomography (OCT) for diagnosing and assessing DME.^[17]^ The publication volume of DME peaked at 548 articles by 2021 (Fig. 2A). As illustrated by the polynomial curve fitting in Figure 2C (y = 0.0065 × 3 − 38.362 × 2 + 75815x − 5E + 07; *R*^2^ = 0.9332), DME research exhibits an overall upward trajectory, with an anticipated increase in publications this year. Figure 2B demonstrates that Citation Frequency and the h-index demonstrated continuous growth from 1998 to 2018, experiencing a gradual decline after 2018. The peak Citation Frequency years were 2012, 2016, and 2018, with a pinnacle of 15,279 citations in 2018. The average h-index for the field was 22.7, reaching a zenith of 52 in 2010, 2015, and 2016. In summary, these data indicate substantial progress in DME research over the past few decades, particularly from 2005 to 2019.

### 3.2. Country and region analysis

**Figure 3.**
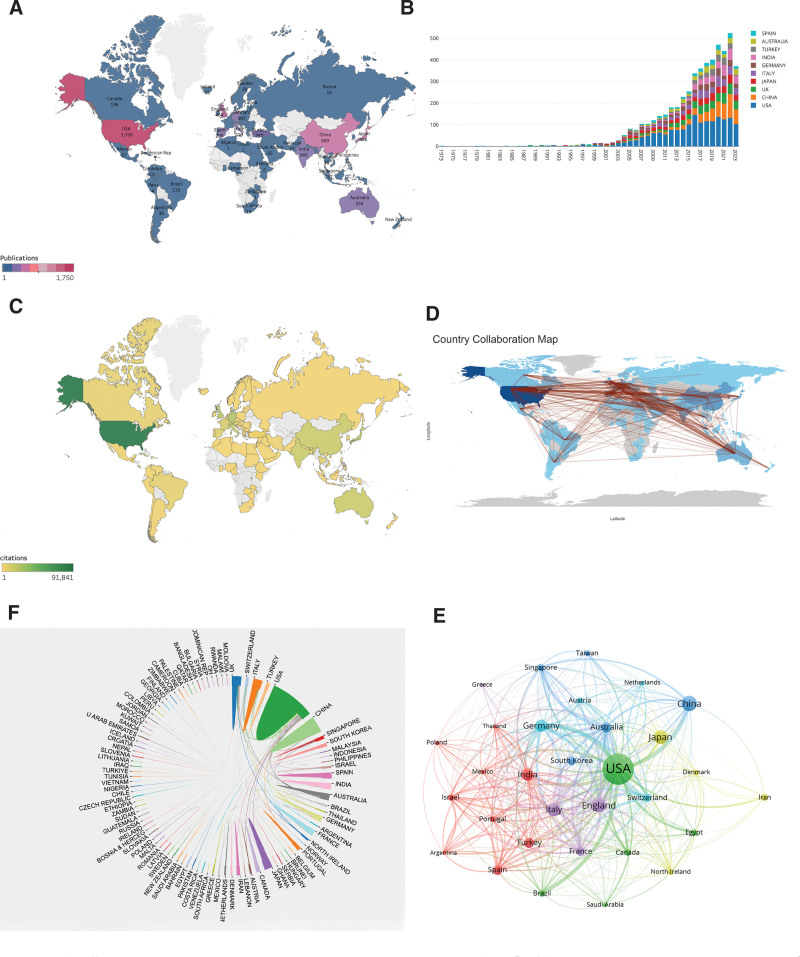
Leading countries/regions in diabetic macular edema. (A) The number of publications in various countries. (B) Annual publication trends of top 10 productive countries. (C) The number of citations in the top 10 productive countries. (D) Country collaboration map. (E) International collaboration between countries. (F) Visual cluster analysis of collaboration among countries.

The overall distribution of DME as a globally recognized topic is evident. From 1973 to 2023, a total of 103 countries/regions worldwide will actively contribute to the publication of papers focused on DME (Fig. 3A). The top 10 countries/regions are predominantly situated in Europe and Asia, collectively constituting approximately 89.08% of the global publications (Fig. 3A and B; Table 1).

**Table 1:** The top 10 active countries/regions.

Rank	Countries/regions	P (%)	CF	h-index	Total link strength
1	USA	1750 (31.12)	91,841	135	1127
2	China	509 (9.05)	13,091	50	244
3	England	464 (8.25)	16,987	57	581
4	Japan	446 (7.93)	16,631	61	152
5	Italy	410 (7.29)	17,708	57	494
6	Germany	347 (6.17)	20,976	65	549
7	India	345 (6.13)	13,003	42	383
8	Turkey	257 (4.57)	3244	29	152
9	Australia	254 (4.52)	12,877	46	510
10	Spain	228 (4.05)	6973	39	291

CF = citation frequency, P = publications.

The United States emerged as the leading contributor, with the highest number of publications (n = 1750), followed by China (n = 509), the United Kingdom (n = 464), Japan (n = 446), Italy (n = 410), and Germany (n = 347) (Fig. 3A). Regarding citations, the United States had the most significant impact, accumulating a total of 91,841 citations, followed by Germany (20,976 citations), Italy (17,708 citations), the United Kingdom (16,987 citations), Japan (16,631 citations), and China (13,091 citations) (Fig. 3C; Table 1). Additionally, the United States exhibited the highest h-index (h-index = 135), followed by Germany (h-index = 65), Japan (h-index = 61), the United Kingdom (h-index = 57), Italy (h-index = 57), and China (h-index = 50). These data underscore the influential roles of the United States, China, the United Kingdom, Japan, Italy, and Germany in the field of DME research.

Figure 3B delineates the annual publication trends in these countries/regions from 1973 to 2023. Notably, between 2013 and 2022, China demonstrated the highest annual growth rate in publications, signifying the escalating enthusiasm and engagement of Chinese researchers in this field. Figure 3D and E reveal robust collaboration among countries in DME research. The United States had a collaborative impact, boasting a total connection strength of 1127, followed by the United Kingdom (581), Germany (549), Australia (510), and Italy (494) (Table 1). While collaboration is extensive in various countries/regions, the thickness of the connections (Fig. 3F) highlights particularly close collaboration with China, followed by Germany, despite China’s high rankings in terms of publication quantity, citation frequency, and h-index. In terms of the h-index, the international collaboration ranking was relatively low, with a total connection strength of 244.

### 3.3. Author and institutional analysis

In total, 18,564 authors affiliated with 5216 institutions contributed to the publication of DME-related articles. The top 10 institutions in DME research output are detailed in Table 2. Johns Hopkins University stands out with the highest number of published papers (n = 211), followed by the University of London (n = 207), Harvard University (n = 188), and Johns Hopkins University School of Medicine (n = 186). Johns Hopkins University leads not only in paper count but also in total citation count and h-index (cited 19,491 times; h-index = 64). Following closely are Johns Hopkins University School of Medicine (cited 17,710 times; h-index = 61) and Harvard University (cited 15,008 times; h-index = 56). The number of papers, citation frequency, and h-index of research institutions serve as valuable metrics to represent their research capabilities in the field.

**Table 2:** The top 10 active institutions.

Rank	Institution	Countries/Regions	P	CF	h-index
1	Johns Hopkins University	USA	211	19,491	64
2	University of London	England	207	9558	44
3	Harvard University	USA	188	15,008	56
4	Johns Hopkins Medicine	USA	186	17,710	61
5	University of California System	USA	182	13,815	50
6	University College London	England	179	5982	38
7	Harvard Medical School	USA	157	12,487	50
8	Moorfields Eye Hospital NHS Foundation Trust	England	148	5467	36
9	University of Sydney	Australia	140	9907	38
10	Udice French Research Universities	France	137	7982	42

**Figure 4.**
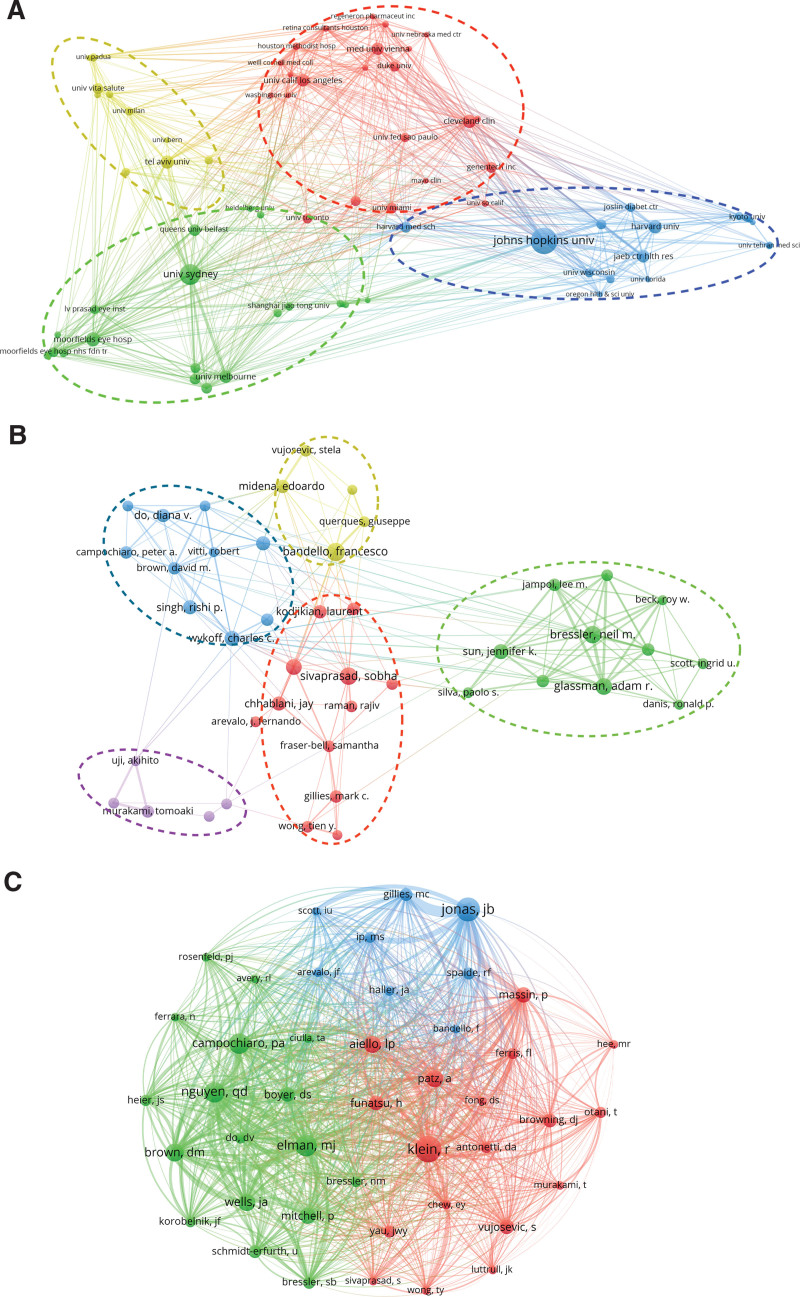
Visualization of institutes and authors analysis. (A) Cluster analysis of collaboration among institutes. (B) Cluster analysis of collaboration among authors. (C) Co-citation analysis of authors.

An analysis of the collaboration network map among research institutions unveils 4 clusters, with Johns Hopkins University exhibiting the closest collaboration with other institutions (Fig. 4A). Cross-regional and cross-institutional collaborations play a pivotal role in fostering in-depth research in the field of DME.

The most prolific author in the field is Francesco Bandello from Ospedale San Raffaele, with 97 published papers, followed by Sobha Sivaprasad from the Moorfields Eye Hospital NHS Foundation Trust (82 papers) and Neil M. Bressler from Johns Hopkins University (78 papers) (Table 3). Neil M. Bressler from Johns Hopkins University also holds the highest h-index and total citation count (9692 citations; h-index = 46), followed by Adam R. Glassman from the JAEB Health Research Center (8658 citations; h-index = 45), and Lloyd Paul Aiello from Joslin Diabetes Center, Inc. (8952 citations; h-index = 41) (Table 3).

**Table 3:** The top 10 productive authors and co-cited authors.

Rank	Author	Countries/Regions	C	CF	h-index	Co-cited author	Co-citation
1	Bandello F	Italy	97	4561	27	Klein R	2040
2	Bressler NM	USA	84	9692	46	Jonas JB	1765
3	Sivaprasad S	England	82	2058	23	Elman MJ	1451
4	Glassman AR	USA	65	8658	45	Nguyen QD	1408
5	Aiello LP	USA	64	8952	41	Campochiaro PA	1367
6	Loewenstein A	Israel	59	2401	26	Brown DM	1131
7	Wykoff CC	USA	58	1496	21	Aiello LP	1078
8	Schmidt-erfurth U	Austria	58	5540	28	Wells JA	1064
9	Wong TY	China	56	7343	29	Patz A	1048
10	Nguyen QD	USA	54	5104	26	Mitchell P	1014

C = counts.

Collaboration among scholars has become a growing trend that contributes to the development of DME research. Scholars with publication counts equal to or exceeding 25 (43/18,564) were used to construct a co-authorship map (Fig. 4B). This map reveals the existence of 5 clusters in the DME field, primarily centered on the aforementioned authors. Scholars within these clusters demonstrate strong academic connections, with the highest degree of collaboration observed in the cluster led by Neil M. Bressler in New Mexico. However, collaborations outside these clusters remain relatively limited.

When two or more authors are simultaneously cited in one or more papers, a cocitation relationship is formed. Among the 49,219 co-cited authors, 10 had co-citation counts exceeding 1000. Klein R has the highest co-citation count at 2040, followed by Jonas JB at 1765, Elman MJ at 1451, and Nguyen QD at 1408 (Table 3). Utilizing authors with cocitation counts of 400 or more (43/49,219) to construct a cocitation network, 3 clusters were identified (Fig. 4C). The main red cluster consists of 18 members, led by the most co-cited author, Klein R (2040 citations). Additionally, it included 3 major co-cited authors, namely Aiello LP (1078 citations), Patz A (1048 citations), and Mitchell P (1014 citations). The second green cluster included 17 authors, led by Elman MJ (1451 citations), Nguyen QD (1408 citations), Campochiaro PA (1367 citations), Brown DM (1131 citations), and Wells JA (1064 citations). The final blue cluster had only 8 authors, mainly led by Jonas JB (1765 citations).

### 3.4. Journal analysis

In our study, 746 journals significantly contributed to DME research. As detailed in Table 4, more than 160 articles were published in 7 journals, all originating from the United States, except for “Eye” and “Clinical Ophthalmology” from the UK. The journal with the highest number of published papers is “Retina - The Journal of Retinal and Vitreous Medicines” (n = 354), followed by “Ophthalmology” (n = 218) and “Investigative Ophthalmology and Visual Science” (n = 196). “Ophthalmology” not only leads in the paper count but also total citation count and h-index (citations = 31,558; h-index = 90), followed by “Retina – The Journal of Retinal and Vitreous Medicines” (citations = 11,872; h-index = 59), and the “American Journal of Ophthalmology” (citations = 11,014; h-index = 63). Additionally, “Ophthalmology” boasts the highest Impact Factor (IF2022 = 13.7).

**Table 4:** The top 10 productive journals and co-cited journals.

Rank	Journal	C	CF	h-index	IF 2022	Q	Co-cited Journal	Co-citation
1	Retina-the Journal of Retinal and Vitreous Diseases (USA)	354	11,872	59	3.3	Q1	Ophthalmology (USA)	30,993
2	Ophthalmology (USA)	218	31,558	90	13.7	Q1	Investigative Ophthalmology and Visual Science (USA)	13,783
3	Investigative Ophthalmology and Visual Science (USA)	196	9065	55	4.4	Q1	American Journal of Ophthalmology (USA)	13,372
4	Eye (England)	170	3706	35	3.9	Q1	Retina-the Journal of Retinal and Vitreous Diseases (USA)	11,775
5	Graefes Archive for Clinical and Experimental Ophthalmology (USA)	166	4382	31	2.7	Q2	Archives of Ophthalmology (USA)	9676
6	American Journal of Ophthalmology (USA)	164	11,014	63	4.2	Q1	British Journal of Ophthalmology (England)	8010
7	Clinical Ophthalmology (England)	162	2082	26	2.2	Q2	Graefes Archive for Clinical and Experimental Ophthalmology (USA)	4602
8	British Journal of Ophthalmology (England)	157	5764	44	4.1	Q1	Eye (England)	4118
9	European Journal of Ophthalmology (Italy)	151	1569	23	1.7	Q4	Diabetes Care (USA)	3843
10	Acta Ophthalmologica (Denmark)	109	1755	22	3.4	Q2	New England Journal of Medicine (USA)	3594

Q = quartile in category.

**Figure 5.**
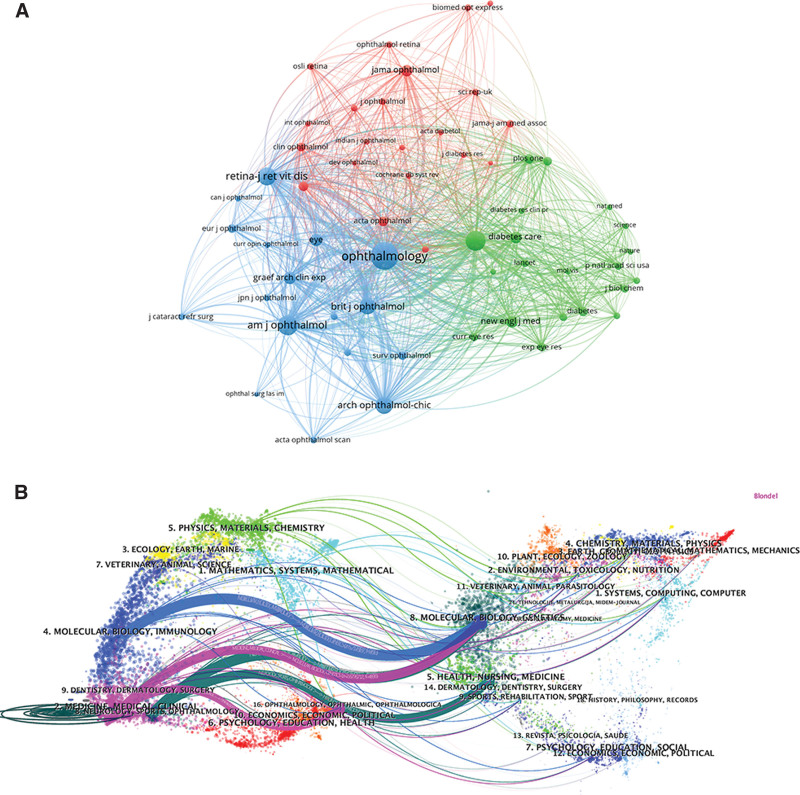
Visualization of journal analysis. (A) Co-citation analysis of journals. (B) The dual-map overlay of papers citing diabetic macular edema research. (The left side is the citing journal, the right side is the cited journal, and the line path represents the citation relationship).

Among the 12,611 co-cited journals, 4 have had co-citation counts exceeding 11,000 (Table 4). “Ophthalmology” stands out as the most frequently co-cited journal, with a total co-citation count of 30,993, followed by “Investigative Ophthalmology and Visual Science” with 13,783 co-citations, and the “American Journal of Ophthalmology” with 13,372 co-citations (Table 4). A co-citation network using 59 journals with co-citation counts equal to or exceeding 530 out of 12,611 journals was constructed. Figure 5A illustrates that “Ophthalmology” maintains active and robust co-citation relationships with related journals such as “Investigative Ophthalmology and Visual Science,” “American Journal of Ophthalmology,” “Retina – Journal of Retinal and Vitreous Diseases,” “Archives of Ophthalmology,” and “British Journal of Ophthalmology.”

The dual-map overlay technique is a novel approach that employs 2 maps simultaneously to visualize the concentration of citations through reference pathways. The left side illustrates the distribution of the major citing journals in DME research in the WoSCC database, whereas the right side shows the distribution of the major cited journals (Fig. 5B). Our dataset, as depicted in Figure 5B, reveals 5 major citation pathways. The citing articles predominantly span fields such as molecular biology, immunology, medicine, clinical studies, neurology, kinematics, and ophthalmology. Conversely, the most-cited articles were clustered in areas such as molecular biology, genetics, ophthalmology, healthcare, and medicine.

As highlighted in Figure 5B, the landscape of DME incorporates multidisciplinary aspects, including 5. physical, material, and chemical; and 3. Ecology, earth, ocean, 7. veterinary, animal, and scientific, and 1. Mathematics, systems, and mathematics underscore the diverse influence of publications from different fields on citation trends in DME.

### 3.5. Analysis of co-cited references

**Figure 6.**
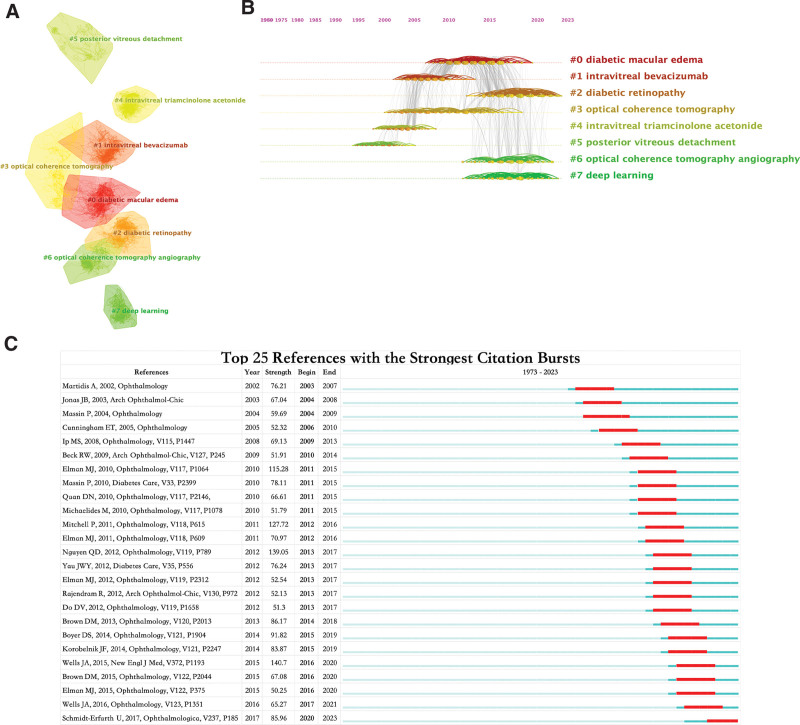
Visualization of co-cited reference analysis. (A) Cluster Analysis of Co-cited References. (B) Timeline distribution of the top 8 clusters. (C) Representative burst references among the top 25 references with the strongest citation bursts.

Conducting a literature co-citation analysis, which involves examining shared citations among references, provides insights into core literature, hot topics, and interdisciplinary connections within the field of DME research. A co-citation clustering view of the references (Fig. 6A), with a modularity Q of 0.7901 and an average silhouette value of 0.8943, was generated. Table 5 presents the data on the 8 major clusters categorized by size, with the understanding that smaller clusters might exhibit lower representativeness than larger clusters. The quality of a cluster is reflected by its silhouette score, which indicates its homogeneity or consistency.

**Table 5:** The top 10 co-cited references cluster analysis.

Cluster ID	Size	Silhouette	Label (LLR)	Mean year	Log (likelihood ratio)
0	271	0.791	diabetic macular edema	2012	7118.28
1	243	0.886	intravitreal bevacizumab	2006	9636.15
2	236	0.804	diabetic retinopathy	2017	7141.16
3	199	0.892	optical coherence tomography	2009	8366.5
4	168	0.951	intravitreal triamcinolone acetonide	2003	7576.82
5	118	0.963	posterior vitreous detachment	1999	877.96
6	115	0.882	optical coherence tomography angiography	2017	16559.21
7	103	0.978	deep learning	2017	5105

Upon examination, most clusters in Table 5 demonstrate high homogeneity, revolving around topics such as “diabetic macular edema,” “intravitreal bevacizumab,” and “diabetic retinopathy.” Notably, the top 10 milestone articles (Table 6) predominantly belonged to Cluster 0, which contained 9 articles, whereas one originated from Cluster 2. The most cited article was Diabetic Retinopathy Clinical Research Network et al,^[18]^ with 456 citations, followed by Nguyen,^[19]^ with 349 citations, and Mitchell P (2011) with 298 citations. These articles are considered significant achievements in their respective research domains.

**Table 6:** The top 10 co-cited references with citation counts.

Rank	Citations	Co-cited reference	Title (Cluster ID)
1	456	Diabetic Retinopathy Clinical Research Network et al., New Engl J Med, V372, P1193	Aflibercept, bevacizumab, or ranibizumab for diabetic macular edema (0)
2	349	Nguyen, Ophthalmology, V119, P789	Ranibizumab for diabetic macular edema: Results from 2 phase III randomized trials: RISE and RIDE (0)
3	298	Mitchell P, 2011, Ophthalmology, V118, P615	The RESTORE study: ranibizumab monotherapy or combined with laser versus laser monotherapy for diabetic macular edema (0)
4	247	Elman, Ophthalmology, V117, P1064	Randomized trial evaluating ranibizumab plus prompt or deferred laser or triamcinolone plus prompt laser for diabetic macular edema (0)
5	245	Boyer, Ophthalmology, V121, P1904	Three-year, randomized, sham-controlled trial of dexamethasone intravitreal implant in patients with diabetic macular edema (0)
6	237	Schmidt-Erfurth, Ophthalmologica, V237, P185	Guidelines for the management of diabetic macular edema by the European society of retina specialists (Euretina) (2)
7	228	Wells JA, 2016, Ophthalmology, V123, P1351	Aflibercept, bevacizumab, or ranibizumab for diabetic macular edema: two-year results from a comparative effectiveness randomized clinical trial (0)
8	224	Korobelnik, Ophthalmology, V121, P2247	Intravitreal aflibercept for diabetic macular edema (0)
9	224	Brown, Ophthalmology, V120, P2013	Long-term outcomes of ranibizumab therapy for diabetic macular edema: The 36-month results from two phase III trials: RISE and RIDE (0)
10	198	Brown, Ophthalmology, V122, P2044	Intravitreal aflibercept for diabetic macular edema: 100-week results from the vista and vivid studies (0)

To further illustrate the temporal evolution of these clusters (Fig. 6B), it becomes evident that “posterior vitreous detachment” and “intravitreal triamcinolone acetonide” constituted early research foci in the field of DME. However, contemporary trends in this field have revolved around “diabetic retinopathy,” “optical coherence tomography angiography,” and “deep learning.”

Figure 6C shows the top 25 references with the most robust citation bursts. Notably, an article by Wells et al published in the New England Journal of Medicine exhibited the highest burst intensity. This study comprehensively evaluated and compared the efficacy and safety of 3 DME treatment methods (Aflibercept, Bevacizumab, and Ranibizumab) in a clinical trial, highlighting the current hotspots in DME research. In addition, in recent years, a review authored by Schmidt-Erfurth U on DME has garnered significant citations. This comprehensive review delves into the various aspects of DME, including its epidemiology, diagnosis, treatment, and prevention.

### 3.6. Keyword analysis

**Figure 7.**
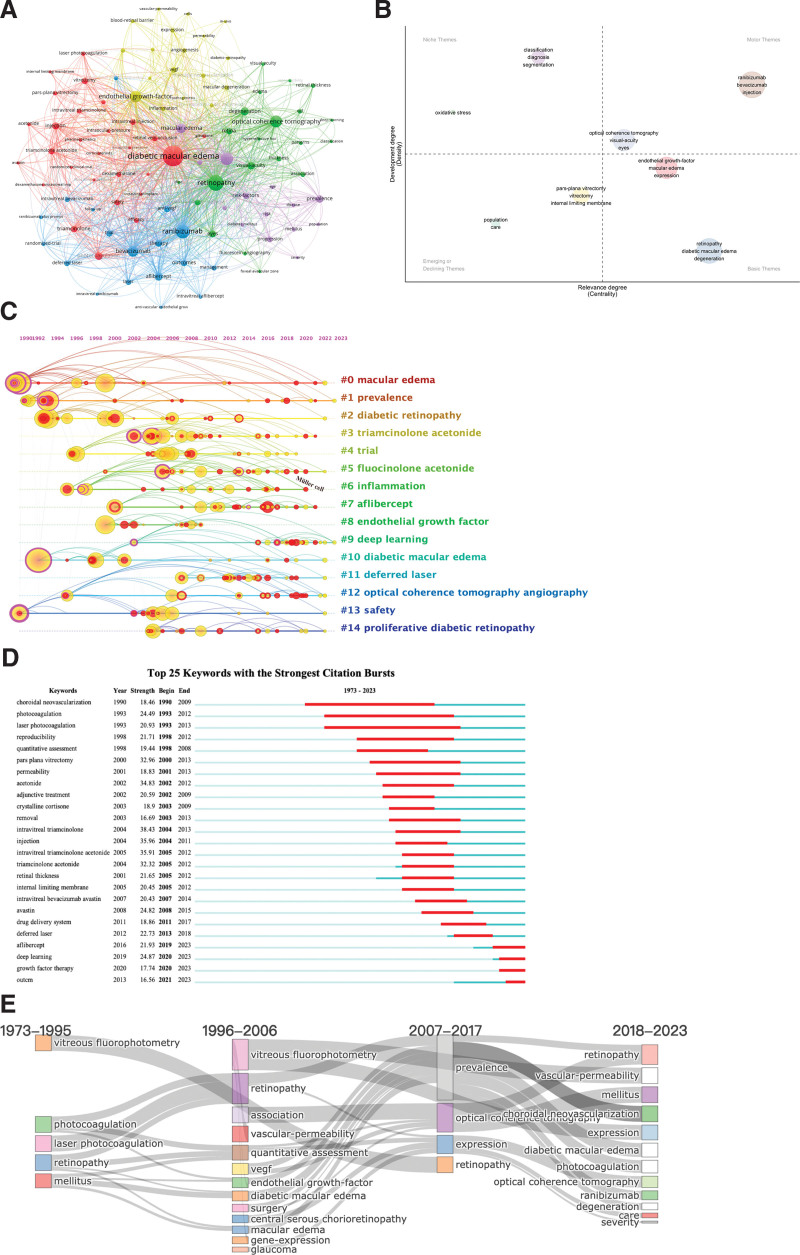
Visualization of keyword analysis. (A) The network map of keywords. (B) Thematic map of keywords. (C) Timeline distribution of cluster analysis of keywords. (D) Representative burst keywords among the top 25 references with the strongest citation bursts. (E) Sankey diagram of the keywords evolution of diabetic macular research.

In total, 9630 keywords were identified within the dataset. By focusing on 99 keywords with a frequency of 80 or higher, a co-occurrence network was constructed (Fig. 7A). Table 7 presents the top 10 keywords ranked by frequency, where “diabetic macular edema” stood out as the core keyword, appearing 2664 times with a total link strength of 12,006.

**Table 7:** The top 10 keywords in the occurrence frequency.

Rank	Keyword	Occurrence	Total link strength
1	diabetic macular edema	2664	12,006
2	retinopathy	1607	7773
3	diabetic retinopathy	1255	7185
4	ranibizumab	1038	5147
5	optical coherence tomography	975	4571
6	endothelial growth factor	915	5202
7	macular edema	791	4963
8	bevacizumab	653	3323
9	visual acuity	477	3025
10	degeneration	470	2249

The thematic map (Fig. 7B) further delineates the research directions, with themes such as Ranibizumab, Bevacizumab, injections, and Optical Coherence Tomography prominently positioned in the upper-right corner. This positioning underscores the significance of notable advancements in these specific areas. Conversely, topics such as Pars Plana Vitrectomy, Vitrectomy, and Inner Limiting Membrane are situated in the lower-left corner, indicating their status as either emerging or declining themes.

CiteSpace was utilized to conduct keyword-clustering analysis, unveiling 23 clusters with an average silhouette value of 0.9507 and a modularity Q of 0.8102. In Table 8, the top 10 clusters based on K-values were present, encompassing themes such as macular edema, prevalence, diabetic retinopathy, intravitreal injections, and trials.

**Table 8:** The top 10 largest clusters of keywords analysis.

Cluster ID	Size	Silhouette	Mean year	Top term	Log (likelihood ratio)
0	29	0.935	1999	macular edema	200.73
1	27	0.986	2002	prevalence	134.84
2	23	0.917	2000	diabetic retinopathy	228.03
3	22	1.000	2009	triamcinolone acetonide	182.37
4	22	0.950	2007	trial	98.64
5	20	1.000	2012	fluocinolone acetonide	86.05
6	20	0.994	2009	inflammation	55.95
7	19	0.948	2013	aflibercept	147.05
8	18	0.934	2003	endothelial growth factor	182.17
9	18	0.955	2018	deep learning	215.1

Furthermore, a timeline view of keyword clusters was created to depict the chronological evolution of DME research (Fig. 7C). The cluster timeline showed that macular edema, prevalence, and diabetic retinopathy represented the earliest and most extensively studied topics among the 10 clusters. Recent investigations have honed keywords such as Ranibizumab, Fluocinolone, Aflibercept, and Deep Learning, signaling contemporary trends in DME research.

Finally, a keyword burst analysis was performed, and Figure 7D displays the top 25 bursting keywords. Intravitreal triamcinolone and injections exhibited the highest burst intensities, primarily reflecting the treatment modalities for DME. Additionally, newly identified keywords, such as Deep Learning, Growth Factor Therapy, and Outcomes, signify recent advancements in the diagnosis, treatment, and clinical research landscape of DME.

A thematic evolution analysis of the keywords, visually represented in Figure 7B and E, was conducted. The findings indicate that the initial phases of DME research predominantly centered around Vitreous Fluorophotometry and Laser Photocoagulation. However, with progress in research, there has been a notable shift in focus towards topics such as Growth Factors, Optical Coherence Tomography, Ranibizumab, Aflibercept, and Deep Learning.

## 4. Discussion

### 4.1. Milestone article analysis

**Figure 8.**
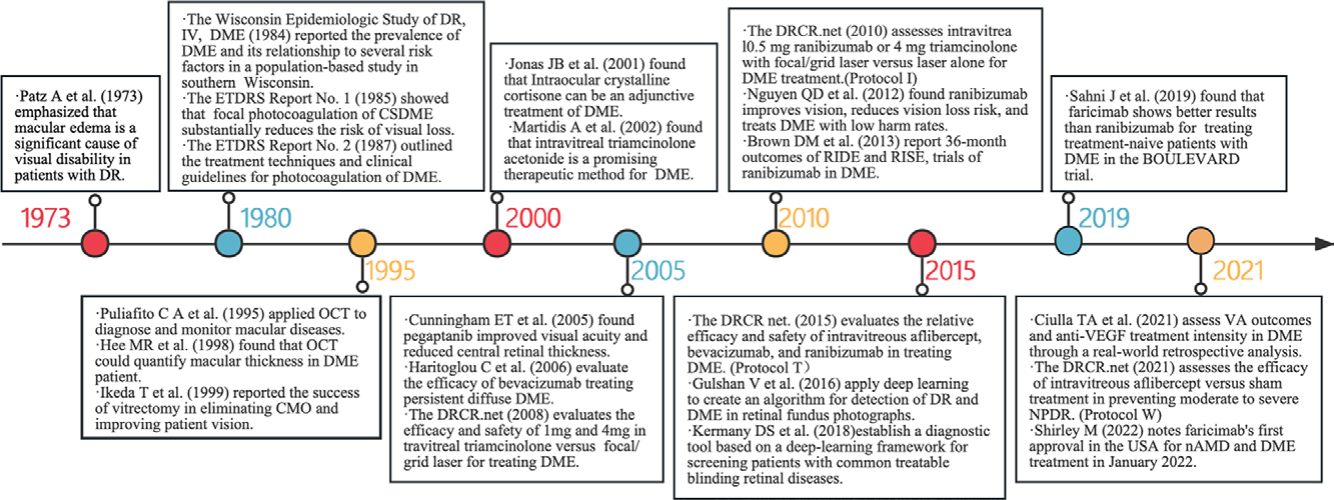
Timeline of significant achievements in diabetic macular edema research.

This study conducts a bibliometric analysis to identify the primary knowledge domains and emerging trends in DME research. We traced the development of DME studies over time, pinpointing key milestone articles that represent significant shifts in the field’s trajectory (Fig. 8). Our milestone analysis begins in 1973, a crucial year in DME research due to Patz et al’s initial use of the term “diabetic macular edema.”^[20]^ This year signifies the starting point of focused academic exploration into DME, setting a benchmark for subsequent studies and marking the dawn of a new research era. Consequently, we utilize 1973 as the foundation from which we track the field’s progression and shifts in focus. In the 1980s, Klein et al conducted a comprehensive epidemiological study of DME in Wisconsin, USA.^[21]^ The Early Treatment Diabetic Retinopathy Study (ETDRS) conducted a series of studies^[22,23]^ during the same period, systematically evaluating the efficacy of laser therapy for DME and establishing fundamental principles and methods for its application. In 1995, the introduction of OCT in clinical ophthalmology marked a transformative moment,^[24]^ ushering in a new era of DME research. Subsequent therapeutic approaches, such as vitrectomy and intravitreal injection of corticosteroids, have been introduced for DME treatment.^[25–28]^

The early 2000s witnessed the emergence of anti-VEGF drugs, including Pegaptanib, Bevacizumab, Ranibizumab, and Aflibercept, as novel therapeutic options for DME.^[29,30]^ This paradigm shift, which replaced traditional laser therapy, has demonstrated significant efficacy and signifies a pivotal juncture in DME management. The influential role of large-scale research groups, such as the Diabetic Retinopathy Clinical Research Network (DRCR.net), in shaping DME treatment strategies has become evident through landmark studies,^[19,28,31,32]^ like Protocol I^[33]^ released in 2010 and Protocol T^[18]^ in 2015. These pivotal studies, coupled with a retrospective and updated examination of the ETDRS, have advanced the comprehension and treatment of DME and steered the field toward new paradigms.

Since the mid-2010s, the integration of deep learning has gradually found applications in DME research.^[34,35]^ In 2019, the BOULEVARD trial, led by Sahni J and colleagues, revealed the superior efficacy of faricimab, the first dual-specificity antibody targeting VEGF and angiopoietin-2, over ranibizumab in treating patients with untreated DME.^[36]^ By 2022, faricimab secured initial approval in the United States for treating neovascular age-related macular degeneration or DME.^[37]^

### 4.2. Productivity analysis of countries, institutions, and authors

The United States emerges as the most prolific country, with 5 out of the top 7 institutions originating from the U.S. Johns Hopkins University, boasting 211 published papers, 19,491 citations, and an h-index of 64, stands out as a major representative. The team led by Bressler NM from this institution has made significant contributions to research on retinal diseases, such as age-related macular degeneration and DME.^[38,39]^ Their most-cited article evaluated the safety and efficacy of lampalizumab versus a sham procedure for enlarging Geographic Atrophy due to age-related macular degeneration.^[40]^ In their latest research,^[41]^ they demonstrated that artificial intelligence (AI) can directly estimate the best-corrected visual acuity from fundus photographs in patients with DME without the need for refraction or subjective visual acuity measurements, often achieving accuracy within 1 to 2 lines on an ETDRS chart. They also explore “time in range” as a novel measure of treatment response in DME.^[42]^

Among the top 10 most-cited references, the United States contributed 6. “Aflibercept, bevacizumab, or ranibizumab for diabetic macular edema”^[18]^ (total citations: 1128; IF (2022) = 158.5) is a major representative. This article assessed the relative efficacy and safety of intravitreous aflibercept, bevacizumab, and ranibizumab for the treatment of DME. This study was a key outcome of the PROTOCOL T study conducted by DRCR.net (https://public.jaeb.org/drcröview/home), a clinical research network focused on diabetic retinopathy (DR) and related eye diseases. PROTOCOL T is a randomized multicenter clinical trial aimed at evaluating the efficacy and safety of aflibercept, bevacizumab, or ranibizumab, 3 anti-VEGF drugs, in improving visual acuity in patients with DME. Wells JA is one of the primary authors of this article and has been dedicated to DME research for an extended period. This article describes his most-cited work.

### 4.3. Core journal analysis

It is noteworthy that among the top 10 journals with the highest co-citations, “Ophthalmology” and “Investigative Ophthalmology and Visual Science (Investig. Ophthalmol. Vis. Sci.)” have consistently held the highest positions in terms of both co-citation frequency and impact factor. This indicates that these 2 journals are favored the most by researchers in the field of DME. Scholars from around the world aspire to publish groundbreaking articles in “Ophthalmology” and “Investig. Ophthalmol. Vis. Sci.” These journals showcase cutting-edge research and significant breakthroughs in DME research. In recent years, research on DME has predominantly focused on exploring treatment, management, and diagnostic imaging. For instance, a recent article published in “Ophthalmology” evaluated longitudinal changes in quantitative ultra-widefield angiography parameters and correlated them with functional outcomes and spectral-domain OCT indicators.^[43]^ An article in “Investig. Ophthalmol. Vis. Sci.” discussed the role of Müller cells in the pathological process of DME and the progress in treating DME by targeting Müller cells through gene therapy.^[44]^ The difference lies in “Ophthalmology’s” emphasis on clinical issues in the field of ophthalmology, particularly in disease diagnosis and treatment, while “Investig. Ophthalmol. Vis. Sci.” leans towards basic research in the ophthalmic field, including laboratory studies and scientific advancements.

### 4.4. Research hotspots

Based on the timeline of references, keyword analysis, and VOSviewer, the current research on DME encompasses various aspects, including studies on the prevalence and risk factors of DME, its pathological mechanisms, imaging modalities, treatment strategies, and clinical trials related to DME.

#### 4.4.1. Prevalence and risk factors for DME

Globally, the prevalence of diabetes continues to increase, leading to a corresponding increase in the incidence of Dr DME, a severe complication of diabetic retinopathy, causes irreversible vision loss and poses a significant threat to eyesight. It is gradually becoming one of the primary preventable causes of blindness in the working-age population.^[45]^ Moreover, the presence of DME consumes more healthcare resources, results in a greater economic burden, and leads to deterioration in the quality of life, making it more severe than in patients with diabetes without retinal complications.^[46,47]^ According to data from the International Diabetes Federation, in 2021, 10.5% (537 million people) of adults aged 20 to 79 worldwide had diabetes, and this is projected to increase to 11.3% (643 million people) by 2030 and 12.2% (783.2 million people) by 2045.^[3]^

DR is the only blinding eye disease with a globally increasing age-standardized incidence rate between 1990 and 2020.^[48]^ Research^[2]^ indicates that, in 2020, the prevalence of DR in the global diabetic population was 22.27% (103.12 million people), with a prevalence of clinically significant macular edema (CSME) at 4.07% (18.83 million people). It is estimated that by 2030, the number of people with DR and CSME will increase by 25.9% and 24.8%, respectively, reaching 129.84 million and 23.5 million individuals. By 2045, these numbers are expected to increase by 55.6% and 51.9%, reaching 160.5 million and 28.61 million people with DR and CSME, respectively.^[2]^

Diabetes is a pathological process involving various systemic factors acting in concert. Review studies^[49–51]^ indicate that factors such as diabetes duration, pregnancy, hyperglycemia, hypertension, dyslipidemia, obesity, nephropathy, microalbuminuria, sleep apnea, diabetic retinopathy severity, cataract surgery, genetic factors, anemia, and others influence the occurrence and progression of DME to varying degrees. Owing to the prolonged treatment process, the need for repetitive interventions, and the potential for vision loss, patients may experience significant social and psychological distress. Therefore, identifying and controlling the risk factors is crucial for preventing and slowing the onset and progression of the condition.

#### 4.4.2. Pathological mechanisms of DME

Based on the timeline of keywords and the results of keyword co-occurrence, the pathological mechanisms of DME primarily involve blood-retinal barrier disruption, inflammation, and vascular endothelial growth factor, among others.

DME arises from an imbalance in fluid entry and exit, leading to extracellular edema within or beneath the retina caused by the accumulation of extracellular fluid and intracellular edema resulting from an increase in intracellular fluid.^[52]^ Extracellular edema is directly associated with vascular leakage caused by blood-retinal barrier (BRB) disruption, whereas intracellular edema is closely linked to the accumulation of intracellular fluid because of the impaired drainage function of Müller cells.^[53,54]^ A seminal article (cited 445 times) highlighted that the breakdown of the blood-retinal barrier is the primary pathological change in DME, with locally synthesized vascular endothelial growth factor and pro-inflammatory cytokines being key pathogenic factors for BRB disruption.^[55]^ The guidelines of the European Society of Retina Specialists on the management of DME indicate that anti-VEGF therapy has become the first-line treatment for DME, and anti-inflammatory corticosteroids have also played a role in DME treatment.^[45]^ However, an important review (cited 1049 times) pointed out that although most patients with DME respond well to VEGF treatment, some exhibit poor responsiveness, and long-term injections may lead to drug resistance.^[56]^ To address this issue, a multi-target treatment approach has been proposed, with the development and clinical trials of a new dual-target drug, faricimab, showing promising progress and offering potential improvements in the treatment of DME.

A keyword co-occurrence analysis for Cluster 6 (Fig. 7C) revealed that the “Müller cell” was the most recently emerging keyword in the last 3 years (2020). Müller cells, which are unique glial cells in the retina, play a crucial role in maintaining retinal function and structure.^[57]^ Müller cells produce various cytokines, regulate water-electrolyte transport, transport excess fluid from retinal tissues to the vitreous and retinal vessels, and participate in the formation of the blood-retinal barrier.^[44,52]^ The role of Müller cells in DME pathogenesis is gaining increasing attention, and they may become new targets for DME treatment. Recent findings by Midena et al using retinal imaging and liquid biopsy techniques suggested that the Disorganization of Retinal Inner Layers (DRIL) is associated with severe dysfunction of Müller cells.^[58]^ DRIL serves not only as an imaging biomarker but also as a parameter related to Müller cell-associated visual function. Midena et al discovered that subthreshold micropulse laser therapy may improve the visual function of patients with DME by restoring Müller cell function, thus providing new insights and approaches for the treatment of DME.^[59]^

#### 4.4.3. Imaging of DME

OCT is one of the most significant advances in ophthalmic imaging. It allows noninvasive, high-quality imaging of various layers of the retina and choroid. This technology enables objective qualitative and quantitative assessments, facilitating the early diagnosis of DME, monitoring follow-up, and guiding anti-VEGF retreatments. In keyword cluster #12, a highly cited review (cited 875 times) highlights the novel noninvasive OCT Angiography (OCTA) built on the OCT platform.^[60]^ OCTA provides deep-resolved images of the retinal and choroidal blood flow, surpassing the levels achieved using traditional imaging methods. OCTA can measure vessel density and detect changes in DR, such as nonperfusion areas, microaneurysms, intraretinal microvascular abnormalities, or neovascularization.^[61]^

Kashani et al reported that OCTA revealed crucial clinical findings, including capillary dilation, impaired perfusion, microaneurysms, capillary remodeling, certain types of intraretinal fluid, and neovascularization.^[62]^ It has also been used to assess various other retinal diseases. Kim et al further discovered that a reduction in capillary density, increased branch complexity, and average vessel diameter were associated with the worsening of Dr^[63]^ Changes in capillary density and morphology are significantly correlated with DME and may have predictive implications.

#### 4.4.4. Treatment of DME

Treatment strategies for DME include intravitreal injection of anti-VEGF drugs, laser photocoagulation, and steroid therapy.

VEGF plays a crucial role in disrupting the BRB, causing vascular leakage, and is a significant factor in the pathological development of DME. Anti-VEGF drugs effectively alleviate vascular leakage, eliminate macular edema, and improve vision. The currently used anti-VEGF drugs include bevacizumab, ranibizumab, aflibercept, and conbercept. Several large clinical studies have demonstrated that anti-VEGF drugs are superior to laser photocoagulation alone in improving visual acuity in patients with Center-Involved DME, making them the first-line treatment options^[33,64–68]^

Laser therapy is one of the main treatment modalities for DME and has been in clinical use for over 30 years. Before the advent of anti-VEGF therapy, focal or grid laser therapy at the macular center was considered the gold standard for DME treatment. Laser therapy can close leaking microaneurysms, induce endothelial repair, seal non-perfused areas, reduce VEGF expression, improve macular microcirculation, suppress vascular leakage, and reduce edema and exudation, ultimately achieving the goal of treating DME.^[45,69]^ The Ranibizumab Monotherapy or Combined with Laser versus Laser Monotherapy for Diabetic Macular Edema (RESTORE) study in 2011 demonstrated the significant superiority of anti-VEGF drug therapy in terms of visual acuity outcomes. Combined with the approval of ranibizumab for DME treatment in 2012, this initiated a new era of DME treatment.^[45,70]^ In the current era of anti-VEGF drugs, laser photocoagulation still has clinical value and can be used as an adjunctive or salvage therapy. Evidence supports delaying laser treatment, specifically for patients with residual edema or recurrent cases after a series of anti-VEGF treatments (3–6 times).^[45,70]^ Guided by fluorescein angiography, focal laser photocoagulation was applied to leaking lesions in the macular area, which correlated with more robust visual outcomes. Subthreshold micropulse laser^[71,72]^ is a relatively new technique based on cellular photostimulation. This can reduce the total laser energy and provide a safe and non-damaging treatment tool. Selective targeting of the retinal pigment epithelium minimizes choroidal retinal scar formation and offers additional options for clinical treatment.

Corticosteroids exert anti-inflammatory effects through various mechanisms, aiding in retinal barrier repair and reducing exudation.^[73–75]^ Many guidelines consider steroids a secondary treatment option for persistent or recurrent edema following anti-VEGF drug therapy.^[75]^ Currently, corticosteroid drugs used for intravitreal injection include non-biodegradable fluocinolone acetonide (Retisert®; Bausch & Lomb Incorporated, Rochester), biodegradable dexamethasone intravitreal implants (Ozurdex®; Allergan Inc, Irvine), and triamcinolone acetonide (TA) (Kenalog®; Bristol Myers Squibb, New York or Trivaris™; Allergan Inc). Retisert®, a nonbiodegradable implant, continuously releases drugs over 36 months. The dexamethasone intravitreal implant is biodegradable, with drug release lasting approximately 3 to 4 months.^[76]^ Compared with traditional intravitreal TA injections (Kenalog® or Trivaris™), Ozurdex® inhibits inflammation through the long-term slow release of dexamethasone, providing a more prolonged and stable anti-inflammatory effect while reducing adverse reactions associated with frequent intraocular injections. Although corticosteroids have been proven to alleviate DME and improve vision in the short term, they are associated with a high risk of cataract surgery and increased intraocular pressure.^[77,78]^

#### 4.4.5. DME-related clinical trials

With the continuous advancement of medical technology and increasing demand for effective disease treatments, researchers have conducted numerous clinical trials in the field of DME. The goal was to explore more efficient and safer treatment strategies to maximally improve the vision and quality of life of these patients. These clinical trials covered various aspects, including comparisons of laser therapy, different drug treatments, and surgical interventions, to gain an in-depth understanding of the effectiveness, side effects, long-term impact, and other factors associated with these treatment methods. The objective was to provide physicians with more precise treatment recommendations, optimize therapeutic strategies, and offer personalized treatment plans and enhanced medical services to patients. These trials are typically collaboratively conducted by multiple institutions, pharmaceutical companies, and research networks. Some key contributors to these trials include the National Eye Institute (NEI), DRCR.net, Regeneron Pharmaceuticals, Inc. (Tarrytown), Bayer HealthCare Pharmaceuticals Inc. (Whippany), Allergan plc (now part of AbbVie Inc., North Chicago), and Genentech, Inc. (a member of the Roche Group, South San Francisco).

The NEI has supported early studies such as the ETDRS, which identified CSME and corresponding laser therapy.^[22,23,79]^ The DRCR.net, supported by the NEI, conducted trials such as Protocol I, Protocol T, and Protocol V, comparing the effectiveness of different drugs and treatment strategies and providing crucial data for the development of treatment guidelines.^[18,80]^ Simultaneously, Regeneron Pharmaceuticals and Bayer HealthCare Pharmaceuticals collaborated to sponsor the VIVID (VEGF Trap-Eye in Vision Impairment Due to DME) and VISTA (Study of Intravitreal Administration of VEGF Trap-Eye in Patients with DME) trials, aiming to evaluate the efficacy of intravitreal aflibercept injections for the treatment of DME.^[67,81]^ Allergan initiated the RESTORE study to investigate the effectiveness and safety of Dexamethasone implants for the treatment of DME.^[82]^ Genentech supported the READ-3 (Ranibizumab for Edema of the Macula in Diabetes – Protocol 3 with high dose) trial, which focused on the efficacy of the ranibizumab injections.^[83]^

In summary, these trials, conducted using rigorous scientific methods, provide a foundation for clinical decision-making and offer more accurate and effective guidance for the treatment of DME. These findings provide scientific evidence for the development of future clinical guidelines.

### 4.5. Emerging research areas

With the deepening exploration of DME research, certain emerging research directions have gradually become topics of interest. An analysis of references and keyword co-occurrence revealed that specific topics experienced a sudden surge in the past 3 years. Classification primarily includes deep learning, artificial intelligence, novel treatment modalities, and biomarkers. Notably, the biomarker category stemmed from keyword co-occurrence analysis results for Cluster 3 over the past 3 years.

The first is deep learning and AI. In recent years, with the rapid advancement in medical imaging and computational capabilities, deep learning and AI have demonstrated tremendous potential in DME research. Currently, deep learning and AI are widely applied to various aspects of DME research, including prediction, early screening, treatment, and management.^[84,85]^ Leveraging deep learning techniques, particularly convolutional neural networks, for in-depth analysis of fundus and OCT images aids in the early screening and precise assessment of DME, significantly enhancing the accuracy and efficiency of lesion detection.^[35,86–89]^ Many researchers have integrated diverse data sources, including fundus images, clinical data, genomic information, and extensive long-term monitoring data, to build predictive models for forecasting the occurrence, progression, and treatment response of DME.^[90–95]^ Personalized treatment plans based on deep learning have also attracted considerable attention. Analyzing large-scale clinical data and establishing models to provide customized, individualized treatment plans for each patient can improve treatment outcomes.^[96–98]^ AI algorithms not only contribute to accurate disease diagnosis but also predict the response of patients with DME to anti-VEGF drug therapy, offering decision support for physicians to personalize treatment and increase opportunities for precision medicine.^[89,99,100]^ Utilizing deep learning and AI, remote monitoring systems can be developed to monitor the retinal conditions of patients in real-time, potentially improving the convenience of diagnosis and treatment as well as alleviating the burden on healthcare systems.^[101,102]^ Although the application of deep learning and AI in the field of DME provides more personalized, accurate, and convenient healthcare services for patients, the advancement of these technologies necessitates addressing a range of challenges, including model interpretability, privacy protection, clinical validation, and formulation of legal regulations.^[103]^

In recent years, the treatment landscape for DME has witnessed a series of innovative approaches, transitioning from traditional therapeutic modalities towards a more personalized and precise phase. Innovations such as long-acting drug delivery systems, the development of novel drugs, gene therapy, stem cell therapy, and the introduction of individualized treatments have provided patients with more personalized and effective choices. Among these, anti-VEGF drugs, which are currently frontline treatments, have demonstrated outstanding therapeutic effects. However, because of the need for frequent injections, the burden of treatment on patients has increased. Therefore, research has begun to explore long-acting treatment methods from the perspective of drug development and novel drug delivery systems to prolong treatment efficacy, reduce treatment frequency, and enhance treatment convenience.^[104–106]^ For instance, the antibody faricimab, which targets Ang2 and VEGF-A receptors, extends the treatment interval to more than 3 months.^[107]^ Simultaneously, the introduction of gene therapy has provided a new perspective on the treatment of DME. Gene therapy holds promise in achieving individualized treatment outcomes by repairing or modulating disease-related genes. The development of gene-editing technologies such as Clustered Regularly Interspaced Short Palindromic Repeats and CRISPR-associated protein 9 enables more precise intervention in patient genomes and provides a more accurate and effective means of treatment.^[108]^ Stem cell therapy has gained widespread attention as an emerging treatment strategy. By harnessing the regenerative and differentiation abilities of stem cells, this therapy aims to promote tissue repair, thereby improving symptoms in patients with DME.^[109,110]^ Research is increasingly focusing on treatment approaches based on individual patient characteristics that encompass factors such as genetic information, pathological features, clinical data, and lifestyle. This involves not only gene-based drug selection but also the integration of artificial intelligence, advanced diagnostic technologies, and comprehensive consideration of the lifestyles of patients to achieve more accurate and targeted disease management.^[89,111,112]^ In future research and practice, further exploration of the potential of personalized treatment will offer more possibilities to enhance patient treatment experiences and outcomes.

Biomarker research in the field of DME is gradually gaining prominence and offers new prospects for personalized treatment and disease management. Biomarkers are measurable indicators that reflect biological state, disease progression, and treatment response. Their application in DME holds promise for providing support for more precise diagnostic and therapeutic strategies. Through in-depth studies of genetic, proteomic, metabolic, and imaging biomarkers, a more comprehensive understanding of the individual differences among patients can be achieved. This aids in distinguishing the different subtypes of DME, predicting disease progression, and facilitating more personalized and precise medical diagnosis and treatment, ultimately offering more effective treatment strategies for patients with DME. Cho et al^[113]^ detected the abnormal expression of certain miRNAs in the aqueous humor of patients with DME, which correlated with cytokine expression. Thus, miRNAs may serve as biomarkers for the diagnosis and treatment of DME. Chan et al^[114]^ predicted the response to anti-VEGF therapy by examining the expression profile of microRNAs in the tears of patients with DME, potentially providing more accurate guidance for clinical treatment. In recent years, with the continuous advancement of medical imaging technology, imaging biomarkers such as OCT and OCTA have gained attention in DME. By combining high-resolution imaging techniques such as OCT and OCTA, researchers can observe and quantify retinal structures and vascular conditions, including retinal edema, exudation, retinal nerve fiber layer thickness, hyperreflective dots, and vascular leakage and dilation. This enhances our understanding of the pathophysiological processes underlying DME. Dimitriou et al^[115]^ found that specific morphological features in retinal imaging, such as retinal edema, bleeding, and exudation, correlated with laboratory parameters, such as blood biochemical indicators, potentially elucidating the pathophysiology of DME and its correlation with specific clinical symptom development. Studies^[58]^ propose that DRIL in eyes with DME is predominantly linked to severe dysfunction of Müller cells. Furthermore, alterations in DRIL are indicative of future changes in visual acuity.^[58,116]^ Huang et al^[117]^ discovered that OCT biomarkers, including subretinal fluid, central foveal thickness, and hyperreflective foci, could help predict the treatment response of patients with DME to anti-VEGF therapy, further promoting individualized treatment. Biomarker research not only enhances our in-depth understanding of DME but also lays the foundation for precision medicine and personalized treatment. In the future, delving deeper into the potential of these biomarkers combined with clinical data and advanced imaging technologies will further drive the optimization of prevention, diagnosis, and treatment strategies for DME.

This study has several limitations. The search was limited to the WoSCC, potentially omitting relevant studies from other databases, and focused narrowly on DME, possibly excluding a broader array of related research. Industry funding, especially from organizations like DRCR.net, might introduce biases affecting the study’s focus and findings. Additionally, the predominance of research from Western countries, particularly the United States, raises concerns about the representativeness and applicability of the findings globally. The analysis also suggests a potential underrepresentation of studies on patient-centric approaches and the socioeconomic aspects of DME management. Furthermore, while artificial intelligence emerges as a promising area in DME diagnosis and management, its ethical implications and accessibility issues are underexplored, indicating a need for more balanced research that addresses clinical and societal challenges.

## 5. Conclusion

This comprehensive bibliometric analysis has revealed significant insights into the global trends and contributions in DME research. Our findings indicate that the United States leads in DME research, contributing significantly to the field in terms of both volume and citations. Notable researchers such as Francesco Bandello, Neil M. Bressler, and Adam R. Glassman have been identified as key contributors, driving advancements in our understanding and treatment of DME. Prominent journals in the field include “Retina – the Journal of Retinal and Vitreous Diseases” and “Ophthalmology,” highlighting where influential research is most often published.

Our analysis has elucidated current hotspots in DME research, including the exploration of prevalence and risk factors, advances in pathological mechanisms, the role of imaging modalities, and the development of novel treatment strategies. Significantly, emerging areas such as the application of deep learning and artificial intelligence, novel treatment modalities, and the identification of biomarkers have been recognized as pivotal future directions in DME research. These findings not only benchmark the current state of research but also outline potential pathways for future investigations.

Moreover, this study underscores the importance of international collaboration and cross-disciplinary approaches in enhancing the quality and impact of DME research. The insights gained from this bibliometric analysis could aid in shaping future research directions, influencing policy-making, and improving clinical practices in managing DME. It is imperative for the research community to continue fostering collaborative efforts and exploring innovative methodologies to advance our understanding and management of diabetic macular edema, ultimately contributing to better patient outcomes worldwide.

## Acknowledgments

The authors sincerely thank the Eye Hospital of the China Academy of Chinese Medical Sciences for their support of this study.

## Author contributions

**Conceptualization:** Yuanyuan Li, Chuanhong Jie, Jianwei Wang.

**Data curation:** Ziqiang Liu, Xiaoyu Hou, Xuqi Bi.

**Methodology:** Weiqiong Zhang, Jingying Wang.

**Software:** Yuanyuan Li, Yu Deng.

**Writing – original draft:** Yuanyuan Li.

**Writing – review & editing:** Chuanhong Jie, Jianwei Wang.
